# Combining Orchardgrass and Alfalfa: Effects of Forage Ratios on In Vitro Rumen Degradation and Fermentation Characteristics of Silage Compared with Hay

**DOI:** 10.3390/ani10010059

**Published:** 2019-12-28

**Authors:** Zhulin Xue, Nan Liu, Yanlu Wang, Hongjian Yang, Yuqi Wei, Philipe Moriel, Elizabeth Palmer, Yingjun Zhang

**Affiliations:** 1 Key Laboratory of Grasslands Management and Utilization, Ministry of Agriculture and Rural Affairs, College of Grassland Science and Technology, China Agricultural University, Beijing 100193, China; xzl2007.ok@163.com (Z.X.); liunan@cau.edu.cn (N.L.); weiyuqi91@126.com (Y.W.); 2 State Key Laboratory of Animal Nutrition, College of Animal Science and Technology, China Agricultural University, Beijing 100193, China; yanluwang@yeah.net (Y.W.); yang_hongjian@sina.com (H.Y.); 3 IFAS-Range Cattle Research and Education Center, University of Florida, Gainesville, FL 32611, USA; pmoriel@ufl.edu (P.M.); e.palmer@ufl.edu (E.P.)

**Keywords:** forage quality, gas production, methane, ruminant

## Abstract

**Simple Summary:**

Forages are an essential portion of ruminant rations to maintain rumen function. Exploring how orchardgrass and alfalfa interact in the rumen is necessary to better understand their feed use potential as both hay and silage. This study evaluated in vitro rumen degradation, fermentation characteristics, and methane production responses to different forage ratios of alfalfa and orchardgrass. The results indicate that dry matter and organic matter degradability and methane production were greater for mixed silages compared to mixed hays. A forage ratio of 50:50 for orchardgrass and alfalfa favor the growth of rumen microorganisms without compromising nutrient digestion and rumen fermentation.

**Abstract:**

This study aimed to investigate the effects of different forage ratios of orchardgrass (*Dactylis glomerata*) and alfalfa (*Medicago sativa*) on in vitro rumen degradation and fermentation characteristics. Orchardgrass and alfalfa were harvested separately and prepared as hay and silage mixtures at ratios of 100:0, 75:25, 50:50, 25:75, and 0:100 (w/w on a dry matter basis) and anaerobically incubated for 48 h with rumen fluid obtained from lactating dairy cows. Fermented residues and cultured fluids were used to determine nutrient degradability, fermentation parameters, and associative effect indices. Increasing the proportion of alfalfa in hay and silage mixtures quadratically increased in vitro organic matter disappearance (IVOMD, up +5.14%) and marginally decreased in vitro neutral detergent fiber disappearance (NDFD, down −1.79%). Meanwhile, increasing the proportion of alfalfa accelerated the rumen fermentation process (e.g., gas production) and remarkably enhanced the growth of rumen microbes as indicated by microbial protein production (MCP, 13.4% increase). Increments of rumen degradability and methane production were more pronounced in silage mixtures than hay mixtures. In combination, a forage ratio of 50:50 for orchardgrass and alfalfa is recommended for both hay and silage in order to improve the feed use potential in ruminants.

## 1. Introduction

Orchardgrass (*Dactylis glomerata*) is one of the most productive cool-season grasses, fairly drought resistant, and is widely distributed in North America, Europe, and East Asia [1]. Previous studies noted that orchardgrass had greater nutritive value than other forages commonly used as feedstuff for livestock, such as bromegrass, tall fescue, and reed canarygrass [2,3]. However, dietary inclusion of orchardgrass hay and silage in steers and sheep should be around 60–75% to avoid negative effects on rumen fermentation [4,5]. Additionally, polyphenol oxidase activity in orchardgrass was able to decrease protein degradation and lipolysis during aerobic ensiling and rumen fermentation [6]. Alfalfa (*Medicago sativa*) has been shown to be rich in crude protein (CP) as well as rapidly degradable protein in the rumen and high inclusion in the diet usually reduces nitrogen (N) utilization and increases urine N excretion [7,8]. However, it is unclear whether a mixture of orchardgrass and alfalfa has the potential to optimize ruminal digestibility and fermentation characteristics through complementary effects of nutrients in the forages.

Digestive interactions may occur in the rumen when ruminants are fed grass-legume mixtures [9,10] and these interactions are due to the diverse impacts on rumen digestion, fermentation, and metabolism efficiency [11]. Positive or negative interactions indicate that the response of ruminants to grass–legume mixtures is either greater or less than the arithmetical calculation obtained from using the responses for grass or legume alone. It would be helpful to determine the proportion of alfalfa at which maximal benefits can be reached in terms of rumen digestibility and fermentation efficiency in order to more precisely formulate rations [12].

The two most common preservation methods of forages, haymaking or ensiling, result in different nutritional compositions, which coincide with changes in ruminal digestibility and fermentation characteristics. For example, alfalfa preserved as hay has less effective protein degradability and digestibility than alfalfa silage, but replacement of alfalfa hay with alfalfa silage did not change ruminal volatile fatty acids (VFAs) or ammonia N [13,14]. Although some studies have separately evaluated the digestibility and fermentation characteristics of silage or hay mixtures [15,16], digestion parameters of silage in comparison with hay mixtures have not been assessed.

The in vitro digestion and gas production method has been widely used to routinely evaluate the associative effects of basal and supplementary feeds with many combinations and is less expensive and time-consuming than in vivo trials [17]. We hypothesized that mixtures of orchardgrass and alfalfa at suitable ratios would increase rumen degradability and fermentation efficiency, while decreasing methane (CH_4_) production compared to alfalfa or orchardgrass alone. Therefore, the objectives of this study were to (1) investigate the associative effects of orchardgrass and alfalfa mixtures on rumen degradation and fermentation characteristics and (2) compare ruminal degradation and fermentation characteristics of hay and silage mixtures.

## 2. Materials and Methods

### 2.1. Experimental Site for Forage Planting

The field component of our experiment was conducted in Dongchengfang County (39°37’ N, 115°51’ E) in Zhuozhou City of Hebei Province of China. In September 2014, orchardgrass (cv. Aba) and alfalfa (cv. WL534) were established as monocultures in 2 field plots (21 × 75 m), each with three replicates. Sowing rate was 15 kg/ha and row spacing was 15 cm for alfalfa and orchardgrass monocultures. Monocultures were grown under the same management practices, with weed-free conditions and no insect pests. The sward soil was a sandy loam with organic matter (OM) content of 13.8 g/kg, total N content of 0.850 g/kg, plant-available phosphorus (P) content of 15.42 mg/kg, available potassium (K) content of 80.12 mg/kg and a pH of 7.80. A total of 75 kg/ha N fertilizer was applied annually in the swards.

### 2.2. Hay and Silage Mixture Preparation

Whole orchardgrass and alfalfa plants were harvested (second cutting) with a reaping hook from the field plots at the jointing and early bloom stages. Ten square areas (1 × 1 m) in each field plot were randomly selected and plants were harvested at a stubble height of 5 cm above ground. The harvested fresh forage was weighed separately and recorded. Representative forage samples (approximately 1 kg) from each square harvest were oven-dried at 65 °C for 48 h to determine the dry matter (DM) yield and chemical composition. Based on the forage DM obtained from four annual harvests, the annual DM yields of orchardgrass and alfalfa averaged 1065 g/m^2^ and 1480 g/m^2^, respectively. The chemical composition of orchardgrass and alfalfa prior to hay making and ensiling was shown in Table 1. After the forage was harvested, representative samples (approximately 20 kg) of each preservation method were divided equally into two subsamples. The first subsample, of both orchardgrass and alfalfa, was air-dried in the sun for 48 h, oven-dried at 65 °C for 48 h, thoroughly mixed to obtain forage ratios of 100:0, 75:25, 50:50, 25:75, and 0:100 (orchardgrass: alfalfa, w/w), and five representative hay samples of each forage ratio were prepared. All of these hay samples were ground in a mill (FW100; Tianjin Taisite Instrument Co., Ltd., Tianjin, China) to pass through a 1.0 mm sieve and stored in plastic bags for chemical analysis and in vitro batch culturing.

The second subsample, of both orchardgrass and alfalfa, was sun-wilted for 2 h until the DM content increased to 400 g/kg and was chopped into 2.5 cm pieces. A microwave oven (M1-L213B, Midea Group Co., Ltd., Foshan, China) was used for the rapid determination of DM content at regular intervals. The wilted forage samples were mixed at ratios of 100:0, 75:25, 50:50, 25:75, and 0:100 (orchardgrass: alfalfa, w/w) based on DM content and were packed into polyethylene silos (1 L capacity) until the total forage weight reached approximately 750 g. Five silo replicates of each forage ratio were prepared, and all silos were sealed with screw lids and kept in the dark at approximately 25 °C. After 40 days of ensiling, a total of 20 g of each silage mixture was homogenized in a blender with 180 mL of distilled water for 1 min. The samples were filtered through a four-layered cheesecloth to prepare the silage extract for determination of ensiling characteristics such as pH, ammonia N, and organic acids, including lactic acid, acetic acid, propionic acid, and butyric acid. Silage mixtures were then dried in a forced-air oven at 65 °C for 48 h and ground in a mill to pass through a 1.0 mm sieve for subsequent analysis.

### 2.3. Rumen Fluid Collection

All experimental procedures used in this study were conducted in accordance with the Institutional Animal Care Committee of China Agricultural University (CAU20171014-1). Rumen fluid was collected from five rumen-cannulated (Type 2c; Bar Diamond Inc., Parma, ID, USA) lactating Holstein dairy cows with a live weight of 530 ± 31.2 kg. Cows were uniformly fed equal rations twice daily at 7:00 a.m. and 7:00 p.m. and provided free access to water. The ration contained 4.0 kg/day of alfalfa hay, 3.5 kg/day of maize silage and 5.5 kg/day of commercial concentrate with a net energy for lactation of 6.69 MJ/kg DM and a CP content of 160 g/kg. Fresh rumen contents (mix of liquid and solid) from different sites inside the rumen were withdrawn from all dairy cows 1 h before the morning feeding with a pair of rubber fabric tongs inserted through the rumen cannula to form one composite sample. The composite sample was squeezed through four layers of surgical gauze to obtain the rumen fluid. Rumen fluid was kept in a thermos flask pre-warmed at 39 °C and immediately transferred to the laboratory. Rumen fluid from the five dairy cows was mixed in equal proportions kept at 39 °C and filled with carbon dioxide (CO_2_) prior to in vitro inoculation.

### 2.4. In Vitro Batch Culturing and Sample Collection

Samples (500 mg) of each forage ratio, of both the hay and silage mixtures, were transferred into 120 mL glass bottles with Hungate stoppers and screw caps, resulting in a total of 50 samples in glass bottles (5 forage ratios × 2 preservation methods × 5 fermentation bottles). Fifty mL of fresh buffer solution at a pH of 6.85 [18] and 25 mL of filtered rumen fluid were mixed in each bottle, which were all purged with nitrogen gas (N_2_) to maintain anaerobic conditions. Thereafter, each bottle was connected to an automated trace gas recording instrument (AGRS-III) [19] to continuously record the cumulative gas production (GP). Five bottles without forage samples were incubated as blanks. Simultaneously, an additional 30 glass bottles (5 forage ratios × 2 preservation methods × 3 fermentation bottles) prepared in the same manner were separately connected to pre-emptied air bags that collected all of the fermentation gases for subsequent determination and calculation. Three fermentations without samples were also included as blanks. All bottles were incubated at 39 °C for 48 h, and these batch cultures were repeated in 3 experimental runs. Two hundred and forty bottles (5 forage ratios × 2 preservation methods × 5 fermentation bottles × 3 experimental replicates + 5 forage ratios × 2 preservation methods × 3 fermentation bottles × 3 experimental replicates) plus 8 bottles in each replicate that were used as blanks (rumen fluid + buffer) were examined in this experiment.

After incubation, the remaining contents in each bottle were separately filtered through preweighed nylon bags (8 × 12 cm, 42 μm pore size). The filtrate was sampled for the determination of ammonia N, microbial protein production (MCP) and volatile fatty acids (VFAs). The nylon bags were thoroughly rinsed and dried at 65 °C for 48 h to a constant weight (the difference between two consecutive weights was no more than 0.20 mg per g) to determine in vitro dry matter disappearance (IVDMD), in vitro organic matter disappearance (IVOMD), in vitro neutral detergent fiber disappearance (NDFD) and in vitro acid detergent fiber disappearance (ADFD) after the chemical analysis of residues remaining in the bags.

### 2.5. Chemical Analysis

Determination of DM, OM, ether extract (EE), CP, and calcium (Ca), K, and magnesium (Mg) followed the methods of the Association of Official Analytic Chemists (AOAC) [20]. Following the method of Van Soest et al. [21], the contents of neutral detergent fiber (NDF), acid detergent fiber (ADF), and acid detergent lignin (ADL) were determined using a Fiber Analyzer (ANKOM Technology Corporation, Fairport, NY, USA) and expressed including residual ash. The concentrations of ammonia N and organic acids, including lactic acid, acetic acid, propionic acid, and butyric acid, in the silage extract were determined according to the method described by Li and Meng [22].

For each cultured fluid, the concentrations of ammonia N [23] and MCP [24] were determined. The determination of VFAs was conducted according to the method described by Cui et al. [25]. Briefly, the filtrate of each cultured fluid kept in polypropylene tubes (10 mL) was centrifuged at 3500× *g* for 15 min at 4 °C and 1.0 mL of the supernatant sample was mixed with 0.3 mL of 250 g/L orthophosphoric acid solution into a polypropylene microtube for 30 min. Thereafter, the mixture was cooled at 4 °C for 2 h and centrifuged at 15,000× *g* for 10 min at 4 °C. The supernatants were kept at −20 °C for later VFAs analysis. Methane, CO_2_, and hydrogen gas (H_2_) produced per kg OM digested were measured with a gas chromatograph according to the method described by Cui et al. [25].

### 2.6. Calculations

Flieg’s score was calculated according to the method of Kilic [26], as shown in Equation (1).

(1) Flieg’s score = 220 + (2 × % DM −15)−40 × pH,

The values at 81–100, 61–80, 41–60, 21–40, and 0–20 represented a very good, good, medium, low and poor silage quality, respectively.

Relative forage quality (RFQ) was calculated according to the method of Moore and Undersander [27], as shown in Equation (2).

(2) RFQ = DMI × TDN / 1.32,

where DMI is dry matter intake (% of body weight) and TDN is the total digestible nutrients (% of DM). For the alfalfa and orchardgrass–alfalfa mixtures, TDN and DMI were determined according to the methods described by the NRC [28] and Mertens [29], respectively, with NDFD adjusted according to Oba and Allen [30]. For orchardgrass, TDN and DMI were calculated following the methods of Moore and Undersander [27] and Moore and Kunkle [31], respectively.

Cumulative GP data [GP_(t)_, mL/g DM] at time (t) were exported from the AGRS-III instrument into a Microsoft Excel file and fitted to a nonlinear model [32] by iterative regression analysis, as shown in Equation (3).

(3) $${\mathrm{GP}}_{t}\;=\;A\;\times \;[1-{\;e\;}^{-c\;\times \;(t\;-\;\mathrm{Lag})}],$$

where A represents the asymptotic GP generated at a constant fractional rate (c) per unit time, t is the time of the gas recording, *e* is the base of the natural logarithm, and Lag represents the lag time before GP commences. The average GP rate (AGPR) at half of A was calculated according to the equation of García-Martínez et al. [33], as shown in Equation (4).

(4) $$\mathrm{AGPR}=\frac{A\;\times \;c}{2\;\times \;(\mathrm{Ln}2\;+\;c\;\times \;\mathrm{Lag})},$$

Single-factor associative effects index (SFAEI, %) and multiple-factor associative effects index (MFAEI, %) were calculated according to the methods described by Niderkorn et al. [34] and Wang [35], as shown in Equations (5)–(7).

(5) $$\mathrm{SFAEI}=\frac{\mathrm{observed}\;\mathrm{value}-\mathrm{calculated}\;\mathrm{value}}{\mathrm{calculated}\;\mathrm{value}}\times 100$$

(6) Calculated value = the measured value of sole orchardgrass × the proportion of orchardgrassin mixtures + the measured value of sole alfalfa × the proportion of alfalfa in mixtures

(7) MFAEI = the sum of each single-factor associative effect value ,

In our current study, MFAEI is the sum of the six SFAEI of RFQ, OMD, NDFD, GP_48_, c, and MCP.

### 2.7. Statistical Analysis

Data were analyzed using a randomized complete block design with two preservation methods (hay vs. silage) and five orchardgrass to alfalfa ratios (100:0, 75:25, 50:50, 25:75, and 0:100) for each block. One-way ANOVA was used to determine the linear and quadratic responses to the proportion of alfalfa inclusion on chemical composition and ensiling characteristics of the hay and the silage mixtures, respectively. The in vitro experimental data consisted of five fermentation replicates per forage mixture sample in each of the three experimental runs, for a total of 150 observations of the mixed ratios and preservation methods treatments. After averaging fermentation replicates within an experimental run, the data of 30 observations were subjected to ANOVA using a general linear model in which the fixed effects of preservation methods and mixed ratios of orchardgrass to alfalfa were considered. All statistical analyses were performed using R software (version 3.2.3). The least squares means and standard error (SEM) were calculated, and orthogonal contrasts were performed to examine the linear and quadratic effects of the proportion of alfalfa in hay and silage mixtures. Significance was declared at *p* < 0.05 unless otherwise noted.

## 3. Results

### 3.1. Chemical Composition of Hay and Silage Mixtures

**Figure 1:**
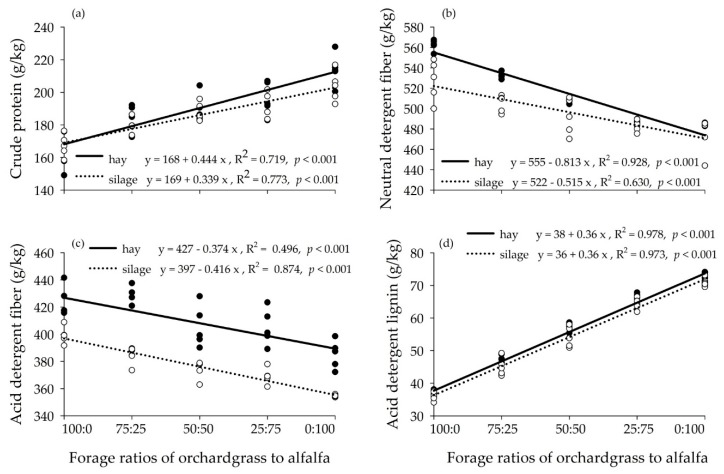

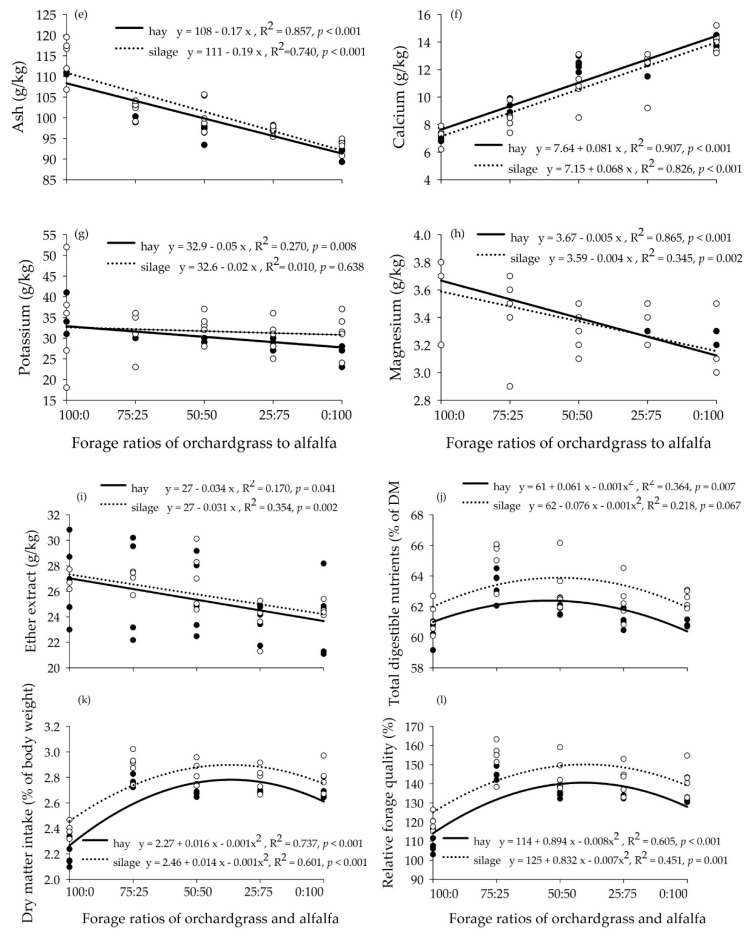
Chemical composition of orchardgrass and alfalfa mixtures preserved as hay and silage: (**a**) crude protein; (**b**) neutral detergent fiber; (**c**) acid detergent fiber; (**d**) acid detergent lignin; (**e**) ash; (**f**) calcium; (**g**) potassium; (**h**) magnesium; (**i**) ether extract; (**j**) total digestible nutrients; (**k**) dry matter intake; (**l**) relative forage quality.

Increasing the proportion of alfalfa increased the contents of CP, ADL, and Ca and decreased NDF, ADF, ash, and Mg in both hay and silage mixtures (Figure 1). Crude protein, NDF, ADF, and ADL were greater in the hay vs. silage mixtures. The TDN, DMI, and RFQ showed a quadratic increase with increasing alfalfa proportions, and these values were reduced in the hay mixtures compared to the silage mixtures (Figure 1).

### 3.2. Ensiling Characteristics of Orchardgrass and Alfalfa Silage Mixtures

The silage pH and the concentrations of ammonia N and acetic acid increased, whereas the concentration of lactic acid decreased as the proportion of alfalfa increased in silage mixtures (Table 2). The concentration of ammonia N in alfalfa was approximately twice that of orchardgrass (11.1 mmol/L and 5.36 mmol/L, respectively). However, the concentration of lactic acid in alfalfa was 355 mmol/L lower than that in orchardgrass (Table 2). According to the Flieg’s scores for silage quality, mixtures of orchardgrass and alfalfa produced excellent quality silages when alfalfa proportion was ≤ 50% (Table 2). The silage mixtures of orchardgrass and alfalfa resulted in higher SFAEI of lactic acid and lower SFAEI of pH, compared with the sole orchardgrass and alfalfa (Table 2).

### 3.3. Degradation Characteristics and Gas Production Kinetics of Hay and Silage Mixtures

**Figure 2:**
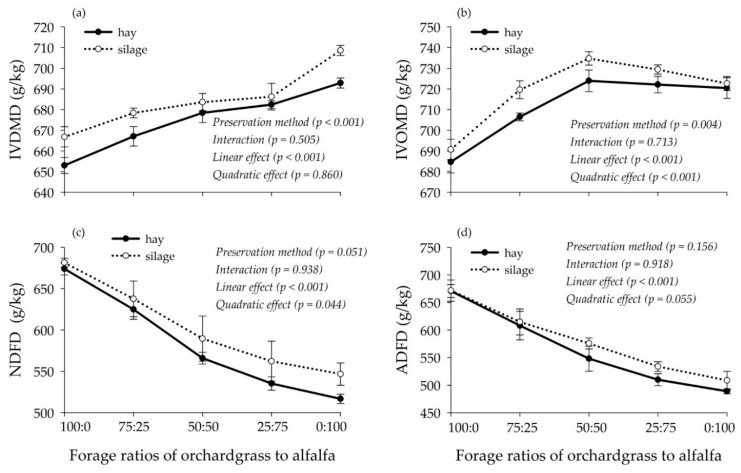
In vitro degradation characteristics of orchardgrass and alfalfa mixtures preserved as hay and silage: (**a**) IVDMD, in vitro dry matter disappearance; (**b**) IVOMD, in vitro organic matter disappearance; (**c**) NDFD, in vitro neutral detergent fiber disappearance; (**d**) ADFD, in vitro acid detergent fiber disappearance. The preservation method indicates whether the forage was preserved as hay or silage, and the interaction indicates the interaction between the preservation method and the ratio of orchardgrass to alfalfa. Orthogonal contrasts were used to examine the linear and quadratic effects of alfalfa inclusion on different hay and silage mixtures. Bars show the mean ± standard error.

The IVDMD increased linearly (Figure 2a, *p* < 0.001), whereas ADFD decreased linearly (Figure 2d, *p* < 0.001) with increasing proportions of alfalfa in hay and silage mixtures. Positive associative effects were observed on IVOMD (Figure 2b, *p* < 0.001), cumulative GP_48_ (Table 3, *p* < 0.05), and asymptotic GP (A) (Table 3, *p* < 0.05); furthermore, the greatest values of these measurements were observed in the 50:50 orchardgrass-alfalfa mixtures. Negative associative effects on NDFD (Figure 2c, *p* < 0.05) were observed when the proportion of alfalfa increased in hay and silage mixtures. The IVDMD (Figure 2a, *p* < 0.001) and IVOMD (Figure 2b, *p* < 0.05) were greater in the silage mixtures than in the hay mixtures, but no effects of preservation method were observed for NDFD (Figure 2c), ADFD (Figure 2d), GP_48_ (Table 3) or other parameters of GP kinetics (Table 3). Positive associative effects were also observed for the fractional GP rate (c) and AGPR as the proportion of alfalfa increased in hay and silage mixtures (*p* < 0.05). Regarding fermentation end-product gases, no changes were observed in CO_2_ or H_2_ production; however, increasing the proportion of alfalfa linearly increased CH_4_ production (*p* < 0.05). Greater CH_4_ production was observed in the silage mixtures than in the hay mixtures (*p* < 0.05; Table 3). No interactions between forage ratios and preservation method were observed on degradation or gas production parameters (*p* > 0.05; Table 3; Figure 2).

### 3.4. Fermentation Characteristics of Hay and Silage Mixtures

Concentration of ammonia N increased linearly (*p* < 0.05) when the proportion of alfalfa increased in hay and silage mixtures (Table 4). A positive associative effect on MCP was detected as the proportion of alfalfa increased (*p* < 0.05). No effects of alfalfa inclusion on the molar proportions of acetate, propionate, butyrate, and isobutyrate were observed (*p* > 0.05). However, the concentrations of total VFAs, valerate and isovalerate increased linearly with increasing alfalfa proportion (*p* < 0.05). No effects of preservation method were observed on these fermentation parameters (*p* > 0.05; Table 4). There were no interactions between forage ratios and preservation method on fermentation parameters (*p* > 0.05; Table 4).

### 3.5. SFAEI and MFAEI of Hay and Silage Mixtures

**Figure 3:**
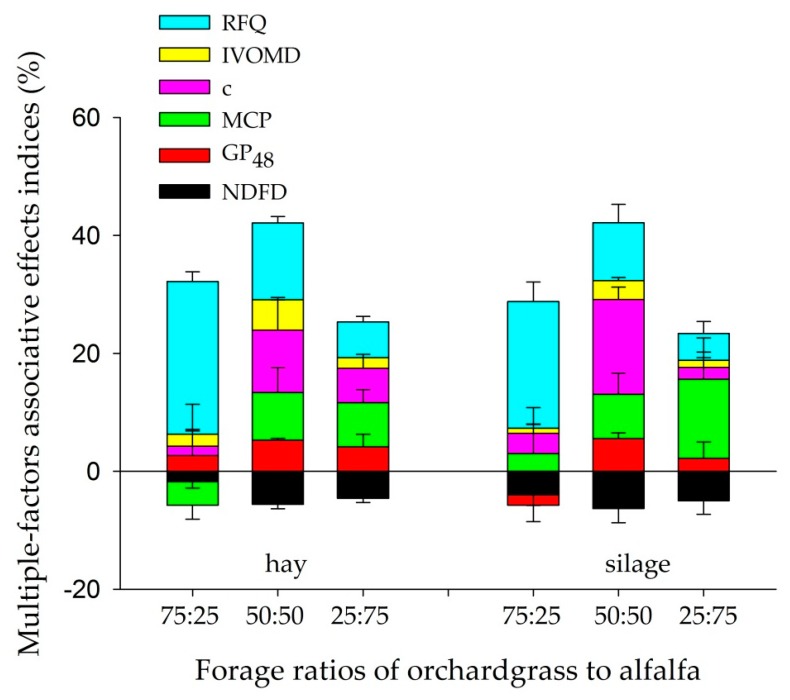
Single-factor associative effects indices (SFAEI, %) and multiple-factors associative effects indices (MFAEI, %) of hay and silage mixtures. RFQ, relative forage quality; IVOMD, in vitro dry matter disappearance; NDFD, in vitro neutral detergent fiber disappearance; c, gas production rate; GP_48_, gas production at 48 h; MCP, microbial protein production. Bars show the mean ± standard error. Single-factor associative effects indices (SFAEI, %) = 100 × [(observed value − calculated value)/calculated value]. Calculated value was determined based on the proportional contribution of sole forage (orchardgrass vs. alfalfa) in the mixture. Positive or negative values indicated positive or negative associative effects of orchardgrass and alfalfa mixtures.

The SFAEI of RFQ, IVOMD, c, MCP, and GP_48_ was positive in both hay and silage mixtures of orchardgrass and alfalfa (Figure 3). The SFAEI of RFQ was greatest at 75:25 orchardgrass to alfalfa hay and silage mixtures. Increasing the proportion of alfalfa increased the SFAEI of IVOMD, GP_48_, c, and MCP by +5.14%, +5.52%, +16.1%, +13.4%, respectively, and decreased the SFAEI of NDFD (−1.79%) when compared with the calculated value. Positive MFAEI was observed in all mixtures, and the highest MFAEI (up +40%) was observed in 50:50 of orchardgrass to alfalfa hay and silage mixtures.

## 4. Discussions

As hypothesized, combining orchardgrass with alfalfa optimized the ruminal degradation and fermentation efficiency through complementary effects of nutrients in forage mixtures and the greatest MFAEI (up about +40%) was obtained at 50:50 of orchardgrass to alfalfa in both hay and silage mixtures. Although mixtures of orchardgrass and alfalfa decreased NDFD, this antagonistic effect was quite little for the total MFAEI, which presumably has been compensated for by synergistic effects of other nutrients. Considering the observed associative effects of mixing orchardgrass and alfalfa, it is possible to optimize forage use efficiency in ruminants.

### 4.1. Chemical Composition of Hay and Silage Mixtures

Greater CP and reduced NDF and ADF contents are usually observed in legumes compared to grasses, due to N fixation from the atmosphere and less cell wall material in legumes [36,37], similar to the current study. A reduction in CP, NDF, and ADF contents were observed in the silage mixtures than in the hay mixtures, probably because a smaller fraction of the protein was broken down into peptides, amino acids, and ammonia and some of the cellulose and hemicellulose were hydrolyzed during the ensiling process [13,38]. Greater values of RFQ were observed in alfalfa-containing mixtures compared with the orchardgrass alone, suggesting that alfalfa inclusion has the potential to improve the overall forage quality of mixtures and the subsequent animal performance [39,40].

### 4.2. Ensiling Characteristics of Orchardgrass and Alfalfa Silage Mixtures

Legumes are usually more difficult to preserve as silage than are grasses due to a reduction in water-soluble carbohydrates and a greater buffering capacity [7]. The results from the current study indicate that alfalfa silage had increased pH and ammonia N concentrations and decreased lactic acid concentrations compared to orchardgrass silage and silage mixtures of orchardgrass and alfalfa showed suitable ensiling characteristics and high associative effects indices. In accordance with previous studies [41,42], silage mixtures could be a feasible way to preserve nutrition and improve fermentation quality through the role of dominated Lactobacillus, compared with the sole silage crops.

### 4.3. Associative Effects on the Degradation Characteristics and Gas Production Kinetics of Hay and Silage Mixtures

The IVDMD linearly increased in response to increasing proportions of alfalfa in the present study due to its high CP and low fiber contents [43]. The IVDMD of silage mixtures was greater than that of hay mixtures, which agrees with previous results that the apparent digestibility of DM and OM in alfalfa silage was greater than that in alfalfa hay [44].

Negative associative effects on NDFD were detected as the proportion of alfalfa increased in mixtures, which is in agreement with other studies [10,45]. These results may be attributed to the antagonistic effects that occur in the fermentation extent and degradation rate of fiber fractions. Similarly, these antagonistic effects were more evident when the proportion of alfalfa was greater than 25% in alfalfa–Caucasian bluestem hay mixtures [46]. The dominant fibrolytic bacteria, that are not proteolytic, require NH_3_ and branched-chain VFAs that are usually derived from active proteolytic bacteria and amino acid fermentation bacteria that do not ferment carbohydrates [47,48]. The degradable and rapidly digested N from legumes compensated for the slow release of N from grasses and coincided with associations and shifts in the ruminal microbial community [15,49] to better degrade the fiber fractions in grass-legume mixtures [46,50].

However, the NDF of legumes is more resistant to degrade than that of grasses because legumes have higher proportions of indigestible NDFs [51] with more complicated structures (e.g., cuticle sheets and vascular bundles) and physicochemical properties (e.g., alkali solubility and ether links), which limit the attachment of fibrolytic bacteria and the enzymatic hydrolysis of forage polysaccharides [37] and decrease the potential for fiber digestion [52]. In the current study, it appears that the positive effect of alfalfa inclusion on NDFD by supplying a rapidly digested N source for fibrolytic bacteria was offset by the negative effect of increased indigestible fiber fractions, subsequently decelerating the fermentation extent and degradation rate of NDF. High levels of fat, starch, and sugars that are rapidly fermented to VFAs can also reduce rumen pH and hence depress the activity of fibrolytic bacteria, decreasing NDFD [53,54,55].

The volume of GP was positively correlated with OM degradability, which is indirectly reflected in the potential of microbes to degrade feed and the extent of microbial fermentation [56]. The current results indicate positive associative effects on cumulative GP and IVOMD when the proportion of alfalfa increased in hay and silage mixtures, implying that complementary digestible nutrients in orchardgrass-alfalfa mixtures provided favorable substrates for microbes, resulting in improved fermentation. The presence of positive associative effects on the asymptotic fractional GP rate (c) but negative associative effects on the time to reach half of the asymptotic GP (half-time) suggests that the inclusion of alfalfa in mixtures accelerated microbial fermentation of digestible components and/or resulted in a shift in the site of digestion of cell contents, including N [9]. In summary, no differences were observed in IVDMD and IVOMD for either hay or silage mixtures containing 50, 75, or 100% alfalfa, however, all mixtures at these proportions had greater IVDMD and IVOMD and reduced NDFD and ADFD values than did mixtures with 25% or 0% alfalfa. If the DM and OM degradability of a mixture needs to be increased, at least 50% alfalfa is required based on the results obtained in this study.

There was no effect on CO_2_ and H_2_ with increasing alfalfa proportions, this could be explained by similar responses in acetate, propionate, and butyrate [57]. The amount of CH_4_ produced per unit of OM digested increased linearly with increasing alfalfa proportion, and the higher nitrogen-free extract (NFE) (44.07% vs. 43.75%) and lower crude fiber (CF) (21.0% vs. 33.93%) in alfalfa compared with orchardgrass could explain the comparatively high CH_4_ production with alfalfa [58,59]. Feedstuffs with higher GP and IVDMD tended to have greater CH_4_ production [60]; this trend is in accordance with the current study where silage mixtures, which had greater IVDMD and IVOMD, had increased CH_4_ production per gram of OM digested than did the hay mixtures.

### 4.4. Associative Effects on the Fermentation Characteristics of Hay and Silage Mixtures

Ammonia N showed a linear increase in cultured fluids as the proportion of alfalfa increased, which was partially due to the proteolysis in alfalfa that occurred during in vitro incubation [61]. Rumen fermentation and microbial protein synthesis proceeded at a lower than maximal rate when the concentrations of ammonia N in the rumen fluid were less than 23.5 mg/dL and 5 mg/dL, respectively [62,63]. In the current study, the concentration of ammonia N in the cultured fluid was above the requirements to maximize MCP synthesis (>5 mg/dL) and thus the sufficient energy should match the synthetic abilities of microorganisms to effectively utilize these N resources [64]. Positive associative effects on MCP were observed as the proportion of alfalfa increased in our study, implying that mixtures of orchardgrass and alfalfa contributed more to the balance of readily degradable carbohydrates and readily degradable protein than did the monocultures, subsequently improving microbial protein synthesis and increasing bacterial N flow and microbial growth.

The concentration of total VFAs linearly increased with increasing proportions of alfalfa, and similarly, increased VFAs production was also observed in sweet sorghum-alfalfa mixtures [15]. In our study, alfalfa inclusion did not change the molar proportions of acetate, propionate, and butyrate, probably because the ruminal microbial community and metabolic pathways by which specific microbes utilize substrates to produce VFAs end-products were not altered [65]. The concentrations of valerate and isovalerate increased linearly with increasing alfalfa proportions, which was in accordance with a previous study [17] showing that the CP level was significantly correlated with valerate and isovalerate production.

## 5. Conclusions

The forage ratio of 50:50 for orchardgrass and alfalfa hay and silage mixtures favored the growth of rumen microorganisms and improved nutrient digestion and ruminal fermentability. Increments of rumen degradability and methane production were more pronounced in silage mixtures than hay mixtures. This study provided potential evidence on how to maximize forage use efficiency in ruminant systems, and further research could be performed to assess the synergistic effects of this practice on agronomic characteristics and ruminant production performance.

## Figures and Tables

**Table 1:** Chemical composition of orchardgrass and alfalfa prior to hay making and ensiling based on dry matter (g/kg).

Item	Orchardgrass	Alfalfa
Dry matter	260	268
Crude protein	167	214
Neutral detergent fiber	562	484
Acid detergent fiber	424	385
Acid detergent lignin	37.0	72.2
Ash	111	92.0
Ether extract	26.8	24.0
Calcium	7.04	14.1
Phosphorus	3.42	3.36
Potassium	33.6	27.8
Magnesium	3.72	3.16

**Table 2:** Ensiling characteristics of orchardgrass and alfalfa silage mixtures.

Item	Forage Ratios of Orchardgrass to Alfalfa					SEM ^2^	*p* ^3^	
	100:0	75:25	50:50	25:75	0:100		Linear	Quadratic
pH	4.50	4.83	4.75	5.03	4.96	0.049	<0.001	<0.001
Ammonia N (mmol/L)	5.36	7.96	9.96	11.2	11.1	0.585	<0.001	<0.001
Lactic acid (mmol/L)	963	998	969	754	608	50.7	0.004	0.005
Acetic acid (mmol/L)	1.58	2.55	7.54	7.88	8.63	0.860	<0.001	<0.001
Propionic acid (mmol/L)	0.38	0.30	0.29	0.31	0.14	0.023	0.002	0.005
Butyric acid (mmol/L)	0.12	0.10	0.13	0.14	0.15	0.008	0.081	0.159
Flieg’s score ^1^	95.6	89.2	87.6	79.3	76.8	1.67	<0.001	<0.001
Single-factor associative effects indices (%) of ensiling characteristics ^4^								
pH	-	4.6	0.5	3.8	-	0.76		
Ammonia N (mmol/L)	-	17.0	20.6	15.4	-	4.09		
Lactic acid (mmol/L)	-	14.2	23.4	8.2	-	5.76		
Acetic acid (mmol/L)	-	−23.7	12.8	14.8	-	10.2		
Propionic acid (mmol/L)	-	−6.4	11.0	51.1	-	11.93		

^1^ Flieg’s score was calculated by means of the pH values and DM contents of the silages: Flieg’s score = 220 + (2 × % DM − 15) − 40 × pH. Flieg’s score: < 20, very bad; 21–40, bad; 41–60, medium; 61–80, good; 81–100, very good. Since there was no difference on DM contents (ranging from 376 g/kg to 383 g/kg) among silage mixtures, the data were not shown. ^2^ SEM, standard error of the mean. ^3^ Linear and quadratic represent the effects of alfalfa inclusion on different silage mixtures. ^4^ Single-factor associative effects indices (SFAEI, %) = 100 × [(observed value − calculated value)/calculated value]. Calculated value was determined based on the proportional contribution of sole forage (orchardgrass vs. alfalfa) in the silage mixture. Positive or negative values indicated positive or negative associative effects of orchardgrass and alfalfa mixtures.

**Table 3:** Gas production kinetics of orchardgrass and alfalfa mixtures preserved as hay and silage.

Item ^1^	Preservation Method	Forage Ratios of Orchardgrass to Alfalfa					SEM ^2^	*p* ^3^		
		100:0	75:25	50:50	25:75	0:100		Preservation Method	Linear	Quadratic
GP_48_ (mL/g DM)	Hay Silage	122 124	128 124	133 136	134 134	131 134	1.1	0.847	<0.001	0.026
GP kinetics										
A (mL/g DM)	Hay Silage	123 124	128 124	134 136	135 134	131 134	1.1	0.837	<0.001	0.025
c	Hay Silage	0.13 0.13	0.14 0.14	0.16 0.16	0.16 0.14	0.15 0.14	0.002	0.165	0.011	0.004
Half-time (h)	Hay Silage	2.71 2.72	2.69 2.66	2.55 2.57	2.60 2.65	2.60 2.65	0.012	0.339	0.005	0.005
AGPR (mL/h)	Hay Silage	23.4 24.2	25.1 24.1	28.4 28.4	29.2 28.5	29.8 29.1	0.37	0.319	<0.001	0.025
Fermentation gas pattern (mL/g OM digested)										
CO_2_	Hay Silage	174 176	176 166	178 178	180 175	176 177	0.8	0.081	0.150	0.675
CH_4_	Hay Silage	31.6 34.6	34.5 37.5	36.2 37.8	36.4 39.0	37.1 39.2	0.62	0.045	0.006	0.287
H_2_	Hay Silage	0.59 2.00	0.36 0.84	0.82 0.94	0.76 0.66	1.51 0.40	0.121	0.476	0.513	0.164

^1^ GP_48_, cumulative gas production at 48 h; A, asymptotic gas production; c, fractional rate of the gas production of ‘A’; half-time, the time when ‘A’ reached its half-maximal value; AGPR, average gas production rate when half of ‘A’ occurred. ^2^ SEM, standard error of the mean. ^3^ The preservation method indicates whether the forage was preserved as hay or silage. Linear and quadratic represent the effects of alfalfa inclusion in the different hay and silage mixtures. Since there were no interactions between the preservation method and the ratios of orchardgrass to alfalfa, we did not present *p*-values on interaction in the Table 3.

**Table 4:** Fermentation characteristics of orchardgrass and alfalfa mixtures preserved as hay and silage.

Item ^1^	Preservation Method	Forage Ratios of Orchardgrass to Alfalfa					SEM ^2^	*p* ^3^		
		100:0	75:25	50:50	25:75	0:100		Preservation method	Linear	Quadratic
Ammonia N (mg/dL)	Hay Silage	20.0 20.0	20.0 20.2	20.4 21.2	21.5 21.7	21.5 21.8	0.31	0.635	0.029	0.996
MCP (μg/mL)	Hay Silage	270 282	276 296	329 315	345 338	338 303	5.2	0.555	<0.001	0.034
Total VFAs (mmol/L)	Hay Silage	110 113	116 110	118 115	117 110	120 119	1.2	0.208	0.049	0.688
VFA pattern (mmol/L)										
Acetate	Hay Silage	71.2 74.5	76.6 70.1	78.4 72.6	77.4 68.8	77.8 76.2	1.10	0.085	0.309	0.809
Propionate	Hay Silage	18.8 17.2	17.6 17.9	17.4 19.5	18.9 17.4	19.0 18.4	0.17	0.297	0.136	0.631
Butyrate	Hay Silage	9.09 9.45	9.76 9.56	9.51 9.82	9.33 10.3	10.2 10.4	0.31	0.620	0.300	0.887
Isobutyrate	Hay Silage	2.14 2.36	2.63 2.34	2.56 2.64	2.14 2.63	2.20 2.83	0.084	0.257	0.900	0.811
Valerate	Hay Silage	2.61 2.80	3.02 2.88	3.10 2.94	2.81 3.25	3.50 3.40	0.093	0.801	0.015	0.710
Isovalerate	Hay Silage	6.04 6.60	6.20 6.76	6.68 7.18	6.54 7.23	7.16 7.99	0.185	0.097	0.024	0.704

^1^ Ammonia N, ammonia nitrogen; MCP, microbial protein production; VFAs, volatile fatty acids. ^2^ SEM, standard error of the mean. ^3^ The preservation method indicates whether the forage was preserved as hay or silage. Linear and quadratic represent the effects of alfalfa inclusion in different hay and silage mixtures. Since there were no interactions between the preservation method and the ratios of orchardgrass to alfalfa, we did not present *p*-values on interaction in the Table 4.
